# Pre- and Post-Natal Maternal Depressive Symptoms in Relation with Infant Frontal Function, Connectivity, and Behaviors

**DOI:** 10.1371/journal.pone.0152991

**Published:** 2016-04-13

**Authors:** Ni Ni Soe, Daniel J. Wen, Joann S. Poh, Yue Li, Birit F. P. Broekman, Helen Chen, Yap Seng Chong, Kenneth Kwek, Seang-Mei Saw, Peter D. Gluckman, Michael J. Meaney, Anne Rifkin-Graboi, Anqi Qiu

**Affiliations:** 1 Department of Biomedical Engineering, National University of Singapore, Singapore, Singapore; 2 Clinical Imaging Research Centre, National University of Singapore, Singapore, Singapore; 3 Singapore Institute for Clinical Sciences, the Agency for Science, Technology and Research, Singapore, Singapore; 4 Department of Obstetrics & Gynecology, Yong Loo Lin School of Medicine, National University of Singapore, National University Health System, Singapore, Singapore; 5 KK Women’s and Children’s Hospital and Duke-National University of Singapore, Singapore, Singapore; 6 Saw Swee Hock School of Public Health, National University of Singapore, Singapore, Singapore; 7 Liggins Institute, University of Auckland, Auckland, New Zealand; 8 Douglas Mental Health University Institute, McGill University, Montréal, Canada; 9 Department of Psychological Medicine, Yong Loo Lin School of Medicine, National University of Singapore, National University Health System, Singapore, Singapore; University of Rennes-1, FRANCE

## Abstract

This study investigated the relationships between pre- and early post-natal maternal depression and their changes with frontal electroencephalogram (EEG) activity and functional connectivity in 6- and 18-month olds, as well as externalizing and internalizing behaviors in 24-month olds (n = 258). Neither prenatal nor postnatal maternal depressive symptoms independently predicted neither the frontal EEG activity nor functional connectivity in 6- and 18-month infants. However, increasing maternal depressive symptoms from the prenatal to postnatal period predicted greater right frontal activity and relative right frontal asymmetry amongst 6-month infants but these finding were not observed amongst 18-month infants after adjusted for post-conceptual age on the EEG visit day. Subsequently increasing maternal depressive symptoms from the prenatal to postnatal period predicted lower right frontal connectivity within 18-month infants but not among 6-month infants after controlling for post-conceptual age on the EEG visit day. These findings were observed in the full sample and the female sample but not in the male sample. Moreover, both prenatal and early postnatal maternal depressive symptoms independently predicted children’s externalizing and internalizing behaviors at 24 months of age. This suggests that the altered frontal functional connectivity in infants born to mothers whose depressive symptomatology increases in the early postnatal period compared to that during pregnancy may reflect a neural basis for the familial transmission of phenotypes associated with mood disorders, particularly in girls.

## Introduction

Exposure to maternal depression has been found to have long-term adverse impacts on neurodevelopment and psychopathology in offspring [1–3]. Both prenatal and postnatal maternal depression associate with an increased risk for emotional [4], behavioral [5], and cognitive problems of offspring [6]. Although most of studies focused on either prenatal or postnatal maternal depression, they have reached a consensus, that is, that greater relative right frontal electroencephalogram (EEG) asymmetry has been suggested to be a neural basis reflecting a vulnerability of offspring to maternal depression [3, 7, 8].

Prenatal maternal depression has been suggested to have a long-term impact on infant physiology through variations in the intrauterine environment [9]. Newborns of prenatally depressed mothers exhibit biochemical profiles similar to those observed in depressed mothers, including elevated cortisol and norepinephrine and lower dopamine and serotonin [10]. Prenatal maternal depression is associated with an increased risk for neurobehavioral, cognitive and socio-emotional problems [11], and depression in the offspring [1, 2, 10]. In addition, limited studies have focused on the relationship between prenatal maternal depression and frontal EEG of the offspring with small numbers of subjects. These studies have suggested that infants born to prenatally depressed mothers had greater right frontal EEG asymmetry than those born to non-depressed mothers [10, 12].

Likewise, postnatal maternal depression promotes forms of parenting [13] that enhance stress reactivity, social withdrawal, and inattention [14–16], which in turn predicts an increased risk for depression and behavioral problems in the offspring [17, 18]. A large body of research consistently found that greater postnatal maternal depressive symptoms predict greater right frontal EEG asymmetry in infants [12, 19] and school-age children [20]. Moreover, children of postnatally depressed mothers showed lower left frontal EEG activity at age of 1- to 3- months [21], through to 6 [22] and 13- to -15 months [19]. Furthermore, a few longitudinal studies have examined the postnatal maternal depression trajectory in relation to children’s frontal EEG asymmetry and behaviors from 14 months to 6.5 years [20] and to children’s social skill from 1 to 36 months [23]. School-aged children of chronically depressed mothers were found to exhibit greater right frontal EEG asymmetry, elevated externalizing behaviors, and decreased social competence [20]. In addition, children’s social skill at age of 4.5 and 6 years are predicted by the changes in maternal depressive symptoms over the course of the first 3 years of postnatal life [23].

Despite strong associations between prenatal and postnatal maternal depression [24], not all mothers may experience the same course of depressive symptoms prenatally and postnatally. Recently, Sandman et al. found that when mothers experienced congruent levels of depressive symptoms during and after pregnancy, even when the levels of symptoms were relatively high and the prenatal and postnatal environments were unfavorable, the offspring increased motor and mental development during the first year of life [25]. This suggests that congruence between prenatal and postnatal environments may be able to prepare the fetus for postnatal life and hence confer an adaptive advantage for critical survival functions during early development. However, limited research has examined the effects of congruence and incongruence between prenatal and postnatal maternal depressive symptoms on frontal EEG function and behaviors of children in the first 2 years of life.

In this study, we first examined whether pre- and early post-natal maternal depressive symptoms independently associate with infants’ frontal EEG activity at 6 and 18 months of age, and internalizing and externalizing behaviors at 24 months of age using a large longitudinal normative Asian sample. We expected to replicate the above-mentioned findings obtained mainly based on clinical samples using the normative Asian sample. Beyond frontal EEG activity, we examined the influence of prenatal and postnatal depression upon frontal functional connectivity (FC). Functional connectivity refers to the functionally integrated relationship among spatially separated brain regions, which is characterized by phase synchronization of EEG signals of two brain region**s** in the infant EEG alpha frequency band (6–9 Hz) [26]. Disrupted frontal connectivity has been suggested as a hallmark of major depressive disorder [27–29]. We therefore anticipated that maternal depressive symptoms might not only influence frontal EEG activity, but also functional connectivity. Nevertheless, we expected the relationship between maternal depressive symptoms and frontal connectivity to be more apparent at later stages of infant development, since the functional integration of brain regions may develop later than that of individual brain structures.

Second, we examined whether the congruence or incongruence of maternal depressive symptoms between pregnancy and early postnatal period, represented by the change of levels of maternal depressive symptoms between pregnancy and postnatal period, influences frontal EEG activity and functional connectivity, as well as internalizing and externalizing behaviors at 24 months of age. Although many studies have examined the relationship between maternal depressive symptoms and frontal EEG activity, less research has examined how the fluctuation of maternal depressive symptoms between pre- and early post-natal period is related to frontal EEG activity. In line with previous research on the disadvantage of the incongruence of prenatal and postnatal environments on early child development [25], we hypothesized that children whose mothers had elevated postnatal maternal depressive symptoms when compared to that during pregnancy may show greater atypical frontal EEG activity and frontal functional connectivity and greater internalizing and externalizing behavioral problems.

Third, we examined the relationship between infant frontal EEG at 6 and 18 months of age and children’s internalizing and externalizing behaviors at 24 months of age. Although frontal EEG activity has been found to associate with internalizing and externalizing behaviors, the relationship between frontal EEG functional connectivity and children’s behaviors has not been explored. We expected that the frontal EEG would be related to children’s behaviors, such that children with higher right frontal EEG activity or lower right frontal connectivity would show more problematic behaviors.

Finally, we examined the above hypotheses in the full sample as well as the female and male samples to investigate possible gender differences in responding to maternal depressive symptoms. The results of this study provide, to our knowledge, the first direct analysis of the link between maternal depressive symptoms, brain frontal organization and children’s behavior in the first 2 years of life.

## Materials and Methods

### Participants

The GUSTO cohort study was approved by the National Healthcare Group Domain Specific Review Board (NHG DSRB) and the Sing Health Centralized Institutional Review Board (CIRB). Written consent was obtained from mothers.

Participants were recruited from a longitudinal Singaporean prospective birth cohort study (Growing Up in Singapore Towards Healthy Outcomes; GUSTO) [30]. Pregnant Asian women were recruited in their first trimester from the National University Hospital (NUH) and KK Women's and Children's Hospital (KKH) in Singapore (n = 1239). The **married couple** were Singapore citizens or Permanent Residents of Chinese, Malay or Indian ethnic background. Socioeconomic status (household income), and other pregnancy measures were extracted from survey questionnaires during pregnancy. Birth outcome and pregnancy measures were obtained from hospital records.

For this study we included only healthy term born infants with a gestational age greater or equal to 37 weeks, birth weight greater than 2500 g, and a 5-min APGAR score equal to or greater than 9 to exclude effects of intra-uterine growth retardation. A total of mother-child dyads (n = 258) from GUSTO met the selection criteria: 6 (n = 174), 18 (n = 157) and 24 (n = 143) months of age, in which 73 infants had EEG data at both 6 and 18 months of age.

### Edinburgh Postnatal Depression Scale

The Edinburgh Postnatal Depression Scale (EPDS) questionnaire was administered to mothers at 26 weeks of pregnancy and 3 months (±1 week) after delivery and used to quantify prenatal and early postnatal levels of maternal depressive symptomatology. The EPDS is a widely used 10-item self-report scale designed as a screening instrument for postnatal depression and has also been well validated for use in prenatal depression [31]. Higher scores indicate a higher intensity of depressive symptoms. In our cohort, the reliability of the EPDS score was 0.82 using Cronbach’s analysis. The fluctuation of maternal depression score was calculated by subtracting prenatal EPDS score from early postnatal EPDS score. Its value greater than 0 indicates a higher postnatal EPDS score than its corresponding prenatal EPDS score.

### Child Behavior Checklist (CBCL)

The CBCL/1.5–5 parental report is a self-administered test of 99 items on emotional, behavioral and social difficulties that characterize preschool children between 1.5 to 5 years of age [32, 33]. The CBCL/1.5–5 exists of seven subscales and also produces internalizing and externalizing problems score. The internalizing problem score is the sum of scores on emotionally reactive, anxious/depressed, somatic complaints, and withdrawn behavior. The externalizing problems score is the sum of scores on attention problems and aggressive behavior. The Cronbach’s alpha’s within our sample ranged from 0.62 to 0.95, which was consistent with Cronbach’s alpha’s reported by Rescorla et al. [34].

### EEG Analysis

The EEG recording procedure is described elsewise [35]. Briefly, the 128-channel Geodesic Sensor Nets connected to a DC-coupled amplifier (Net Amp 300, Electrical Geodesic Inc.) were used to measure brain activity during 40 min of a passive auditory oddball task in infants at 6 months and 18 months of age. During EEG preprocessing, the channels with movement artifacts, flat line and electrode popping artifacts were removed for further analysis (n = 29). For the rest of the EEG channels (n = 99), artifacts, including eye blinks and muscle movements, were identified via visual inspection and removed using EEGLAB toolbox [36]. Finally, 10-minute EEG data from the auditory oddball task for each subject were extracted for spectral power and brain functional network analyses below.

#### Spectrum Power Analysis

**Fig 1:**
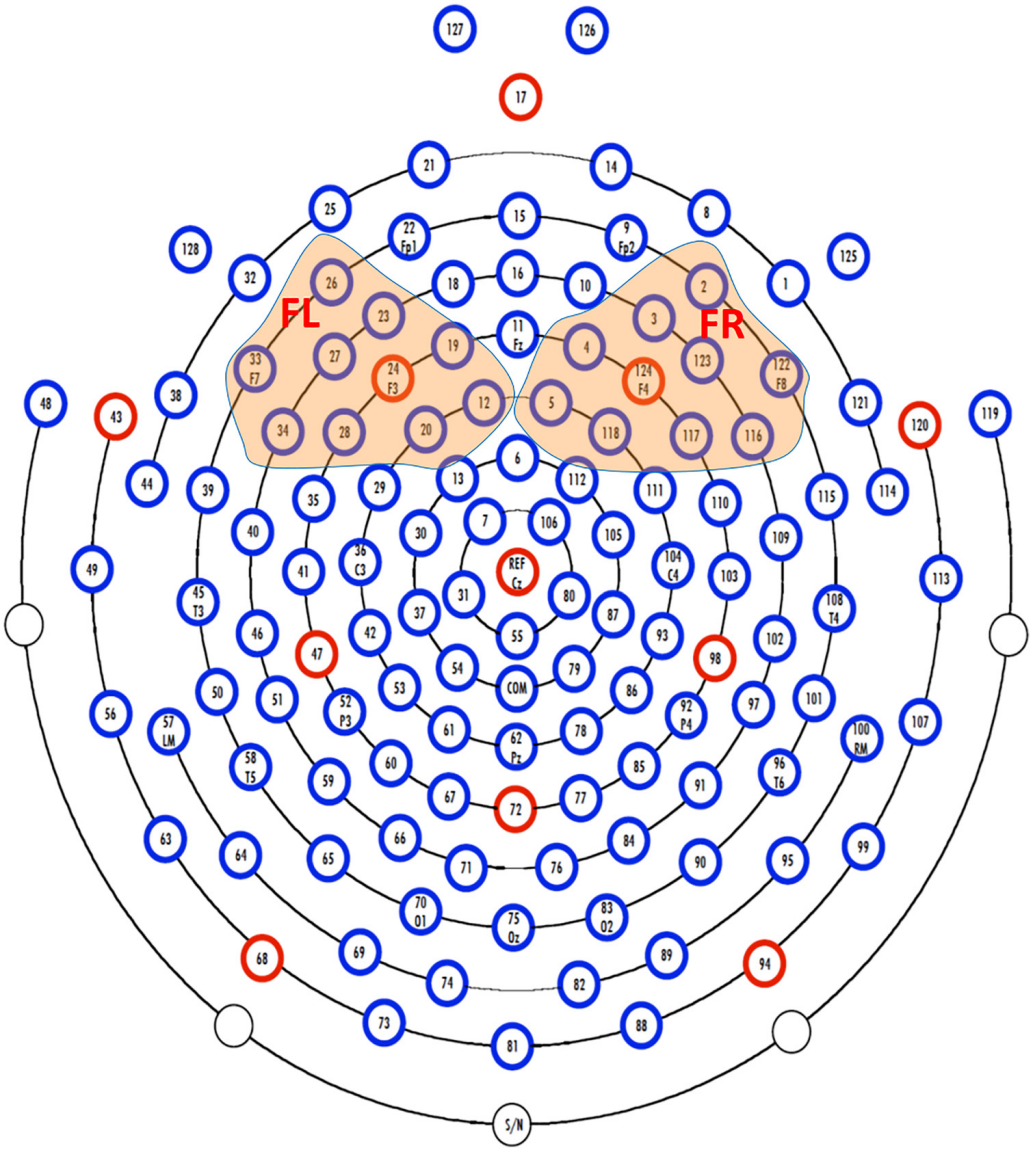
EGI HCGSN128 electrode net. The channels in the frontal left (FL) and right (FR) regions are colored and used in this study.

We computed an absolute power spectrum of each channel ranging in the infant alpha frequency band (6-9Hz) using discrete Fourier transform (DFT) with a Hamming window 2s wide epoch and 50% overlap between epochs. The power spectra were then log-transformed and averaged across the frontal left (FL) channels (12, 19, 20, 23, 24, 26, 27, 28, 33, 34) and frontal right (FR) channels (2, 3, 4, 5, 116, 117, 118, 122,123, 124) (see Fig 1) responses from that scalp region. The greater power spectrum represents lower frontal activity [37]. Frontal power asymmetry (FA) was computed as 2(FR-FL)/ (FR+FL). Its negative value reflects greater relative right frontal activity (rightward asymmetry), while **its** positive value reflects leftward asymmetry.

#### Functional connectivity (FC)

We computed phase synchronization to represent the functional connectivity between two EEG signals in the infant alpha band. Its value ranges from 0, indicating no synchronization, to 1, indicating the greatest synchronization between the two signals. Detailed description of the phase synchronization calculation [29] is widely used in neurophysiology since the analysis can be restricted to certain frequency bands reflecting specific brain rhythms, which allows relating the results to cognitive processes [38].

We computed a functional connectivity matrix (A) whose element (A_ij_) is phase synchronization between the i^th^ and j^th^ channels. We then computed the functional connectivity of the i^th^ channel by averaging A_ij_ over j. Finally, we computed the average functional connectivity over the channels in frontal left (FL) and right (FR) regions to represent FL and FR functional connectivity, respectively. Similarly, asymmetry of the frontal (FA) functional connectivity was computed as 2(FR-FL)/ (FR+FL). Its positive value reflects greater frontal functional connectivity in FR than that in FL (rightward asymmetry), while **its** negative value reflects leftward asymmetry.

### Statistical Analysis

First, we examined the relationships of plausible covariates, including gender, birth-weight, post-conceptual age on the visit day (gestational age + days of life since birth to the visit day), ethnicity, prenatal smoking exposure, and child sleep condition at the time of EEG recording with outcome measures (frontal EEG power, functional connectivity at 6 and 18 months of age, or behavioral scores at 24 months of age). When correlations reached the level of significance (p<0.05), the variables were entered as covariates in the following regression models. Since only one or two mothers had history of prenatal alcohol exposure, this variable was not further used in the following analysis. Note that we did not consider maternal education, maternal age, and birth order as covariates in the below regression analysis to avoid collinearity with monthly household income since monthly household income was highly correlated with maternal education (r = 0.623, p<0.001), maternal age (r = 0.249, p<0.001), and birth order (r = -0.203, p = 0.002).

To examine our first aim, separate regression models were used to evaluate each frontal EEG measure (dependent variable; frontal EEG power: FL, FR, and FA; functional connectivity: FL, FR, and FA), and each behavioral score (dependent variable; internalizing and externalizing score) in relation with pre- or post-natal maternal EPDS score (independent variable). In each regression model, one of the EEG measures or behavioral scores was entered as a dependent variable and prenatal or postnatal EPDS was entered as independent variable. Post-conceptual age on the visit day of EEG was entered as covariate for the EEG measures. Maternal smoking exposure and maternal ethnicity were included as covariates in all regression models for the behavioral outcomes.

To examine our second aim, regression analyses were conducted to examine the associations of the fluctuation of maternal depression (postnatal—prenatal) with frontal EEG at 6 and 18 months of age and children’s internalizing and externalizing behaviors at 24 months of age. The fluctuation of the EPDS score was entered as an independent variable in the regression model. Post-conceptual age on the visit day of EEG was entered as covariate for the EEG measures. The severity of maternal depressive symptoms (average of the prenatal and postnatal EPDS scores) was entered as an additional covariate.

For the third aim, regression analyses were conducted to examine the associations of frontal EEG at 6 and 18 months of age (independent variable) with externalizing and internalizing behavioral scores at 24 months of age (dependent variable). Covariates in these regression analyses included birth weight and post-conceptual age at the EEG visit.

In all the above regression models, prenatal and postnatal EPDS scores were considered as continuous variables. The fluctuation of maternal depressive symptoms between pregnancy and early postnatal period was computed as the subtraction of prenatal EPDS score from the postnatal EPDS score (postnatal—prenatal), which was also a continuous variable in the regression model. We repeated the above analysis when additionally controlling for monthly household income and infant sleep condition at EEG recording time. The monthly household income variable has the value of 1, 2, 3, 4, 5 with the largest value being the highest income and the lowest value being the lowest income. We entered this as continuous variable in the regression model. In each of the above regression analysis, we first entered covariates in the first block of the regression and the main factors in the second block of the regression. The three hypotheses examined using the above regression models were investigated in the entire sample, female and male samples, separately.

## Results

### Demographics

Table 1 lists the demographic information of infants and their mothers. The ranges of prenatal and postnatal EPDS scores are respectively from 0 to 21 and from 0 to 24. The Pearson’s correlation between measures of prenatal and postnatal EPDS scores was 0.600 (r = 0.600; p<0.001, df = 201), however the severity of maternal depressive symptoms was significantly greater during pregnancy than at early postnatal period (t = 3.587, df = 200, p = 0.000). Birth weight and gender were not significantly associated with both pre- and post-natal EPDS scores (p>0.05). However, ethnicity, monthly household income, and prenatal smoking exposure were significantly associated with pre- and post-natal EPDS scores (p<0.05).

**Table 1:** Demographics of the EEG sample at each time point.

Measure	6 months (n = 174)	18 months (n = 157)	Overlap (n = 73)
Gestational age (week), mean (SD)	39.0 (1.0)	38.8 (0.9)	38.9 (1.0)
Birth weight (gram), mean (SD)	3185.7 (380)	3147.9 (354)	3178.4(369)
APGAR score, mean (SD)	9.0 (0.1)	9.0 (0.1)	9.0 (0.1)
Gender, male/female	82/92	77/80	33/40
Birth Order, %			
1^st^ child	44.8	37.6	37.0
2^nd^ child	30.5	31.8	30.1
3^rd^ child	17.8	21.0	23.3
4^th^ child and above	6.9	9.5	9.5
Post-conceptual age on the EEG day (week), mean (SD)	184.5 (6.6)	553.8 (13.7)	
Sleep condition at EEG recording time, % asleep	63.2	26.1	
Prenatal Maternal Depressive symptoms (EPDS), mean (SD)	7.6 (4.4)	7.9 (4.4)	7.6 (4.2)
Postnatal Maternal Depressive symptoms (EPDS), mean (SD)	6.8 (4.8)	6.4 (4.5)	6.6(4.6)
**Severity of Maternal Depressive symptoms, mean (SD)**	**6.9 (4.1)**	**7.1 (4.0)**	**6.8 (3.8)**
**Fluctuation of Maternal Depressive symptoms, mean (SD)**	**-0.63 (4.2)**	**-1.3 (4.1)**	**-0.59 (4.2)**
Maternal Age, mean (SD)	30.5 (5.0)	30.1 (5.6)	30.4 (5.1)
Prenatal smoking exposure, % yes	35.6	47.1	42.5
Prenatal alcohol exposure, % yes	4.6	1.9	2.7
Maternal Education, %			
primary	4.0	4.5	4.1
secondary	20.1	24.8	21.9
gce/ite_ntc	37.9	40.1	38.4
university	33.3	24.2	30.1
postgraduate/others	2.3	3.8	2.7
unreported	2.3	2.5	2.7
Ethnicity, %			
Chinese	54.6	44.6	46.6
Malay	26.4	37.6	35.6
Indian	16.1	13.4	16.4
unreported	2.9	4.4	1.4
Monthly Household Income (S$), %			
≤ 999	2.9	2.5	2.7
1000–1999	10.9	14.6	12.3
2000–3999	27.6	35.0	31.5
4000–5999	24.7	21.0	21.9
≥6000	25.9	21.0	24.7
unreported	8.0	5.9	6.9

Abbreviations: SD—standard deviation; EPDS—Edinburgh Postnatal Depression Scale.

Among the EEG sample, 97 children at 6 months of age and 90 subjects at 18 months of age had CBCL data at 24 months of age. The mean and standard deviation of the CBCL externalizing score were 11.887 ± 7.169 among the 6-month EEG sample and 11.911 ± 7.053 among the 18-month EEG sample. The mean and standard deviation of the CBCL internalizing score were 8.495 ± 7.661 among the 6-month EEG sample and 9.211 ± 8.04 among the 18-month EEG sample.

### Effects of Prenatal and Postnatal Maternal Depressive Symptoms on Infants’ Frontal Function and Behaviors

#### Frontal Activity

Adjusted for post-conceptual age on the EEG visit day, regression analysis did not reveal an association of prenatal or postnatal EPDS with the bilateral frontal activity and its asymmetry in 6-month and 18-month-old infants (Table 2).

**Table 2:** Effects of independent contribution of pre-, early post-natal, severity and fluctuation of maternal depressive symptoms on infants’ EEG at 6 and 18 months and CBCL at 24 months of age in the full sample.

	EEG at 6 month^a^						EEG at 18 month^b^						CBCL behaviors at 24 months ^c^	
	Power			FC			Power			FC				
	FL	FR	FA	FL	FR	FA	FL	FR	FA	FL	FR	FA	externalizing	internalizing
**Prenatal MDS**	0.033	0.061	0.067	0.007	-0.007	-0.008	0.018	-0.03	-0.066	0.128	0.095	-0.088	0.239**	0.332**
**Early postnatal MDS**	-0.045	-0.073	-0.051	-0.004	-0.016	-0.004	-0.033	-0.046	0.023	0.047	-0.048	-0.127	0.304**	0.304**
**Severity of MDS**	0.006	0.010	0.025	-0.037	-0.028	0.020	0.008	-0.025	-0.002	0.120	0.046	-0.126	0.320**	0.377**
**Fluctuations of MDS**	-0.142	-0.238**	-0.205**	-0.025	-0.007	0.020	-0.121	-0.091	0.040	-0.12	-0.196*	-0.047	0.105	0.033

Standardized beta values are listed in the table.

*p <0.05

**p <0.01

^a, b^ Regression models were adjusted for post-conceptual age at the visit date on EEG recording.

^c^ Regression analysis was adjusted for prenatal smoking exposure, and ethnicity.

Abbreviations: MDS–maternal depressive symptoms; FL–frontal left; FR–frontal right; FA–frontal asymmetry; FC–functional connectivity.

#### Frontal Connectivity

Neither prenatal nor postnatal EPDS scores independently associated with bilateral functional connectivity and its asymmetry in 6-month and 18-month-old infants, after adjusting for post-conceptual age on the EEG visit day (Table 2).

#### Externalizing and Internalizing Behaviors

Greater prenatal and postnatal EPDS scores independently predicted higher externalizing and internalizing scores at 24 months of age, after adjusting for maternal ethnicity and prenatal smoke exposure (Table 2).

The above findings remained the same when additionally controlling for monthly household income and sleep condition during the EEG recording in the regression models.

### Effects of Severity of Maternal Depressive Symptoms on Infants’ Frontal Function and Behaviors

#### Frontal Activity

Adjusted for post-conceptual age on the EEG visit day, regression analysis did not reveal an association of severity of maternal depressive symptoms (average of prenatal and postnatal EPDS scores) with the bilateral frontal activity and its asymmetry in 6-month and 18-month-old infants (Table 2).

#### Frontal Connectivity

Severity of maternal depressive symptoms was not associated with bilateral functional connectivity and its asymmetry in 6-month and 18-month-old infants, after adjusting for post-conceptual age on the EEG visit day (Table 2).

#### Externalizing and Internalizing Behaviors

Greater severity of maternal depressive symptoms independently predicted higher externalizing and internalizing scores at 24 months of age, after adjusting for maternal ethnicity and prenatal smoke exposure (Table 2).

The above findings remained the same when additionally controlling for monthly household income and sleep condition during the EEG recording in the regression models.

### Effects of Fluctuation of Maternal Depressive Symptoms on Infants’ Frontal Function and Behaviors

#### Frontal Activity

**Fig 2:**
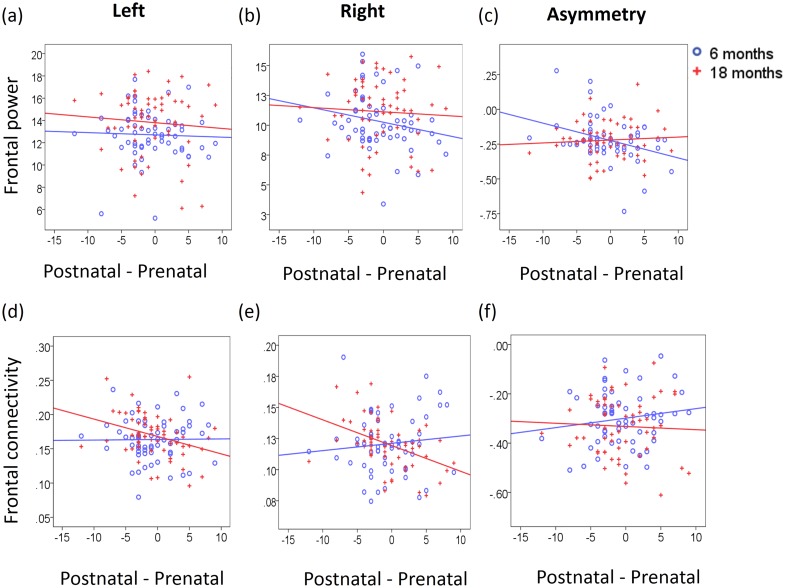
Scatter plots of maternal depressive symptoms and EEG. Panels show scatter plots of the fluctuations of maternal depression from pregnancy to postnatal period with frontal power and frontal connectivity at 6 and 18 months of age.

After adjusting for post-conceptual age on the EEG visit day, greater-postnatal-than-prenatal maternal depressive symptoms were significantly associated with greater right frontal activity (Table 2, Fig 2b) and greater relative right frontal asymmetry in infants at 6 months of age (Table 2, Fig 2c). However, the fluctuation of maternal depression was not associated with the left frontal activity in infants at 6 and 18 months of age, as well as right frontal activity and its asymmetry in infants at 18 months of age (Table 2, Fig 2a–2c).

The above findings remained unchanged after controlling for the severity of maternal depressive symptoms, averaged EPDS score between pregnancy and the early postnatal period, and infant sleep condition during the EEG recording. However, when controlling for monthly household income, regression analysis revealed that greater-postnatal-than-prenatal maternal depressive symptoms were associated with both greater left and right frontal activity at 6 months of age (FL: β = -0.186, p = 0.035, df = 125; FR: β = -0.241, p = 0.005, df = 125). Greater-postnatal-than-prenatal maternal depressive symptoms were marginally associated (FA: β = -0.148, p = 0.098, df = 125) with greater relative right frontal asymmetry of infants at 6 months of age. Nevertheless, the findings for the 18-months EEG data remained unchanged after controlling for monthly household income.

#### Frontal Connectivity

After adjusting for post-conceptual age on the EEG visit day, no significant associations were observed between the EPDS fluctuation and bilateral frontal FC nor its asymmetry in 6 month olds (Table 2, Fig 2d–2f). Likewise, the EPDS fluctuation was not associated with left frontal FC or FC asymmetry in 18 month olds (Table 2, Fig 2d–2f). Interestingly, however, greater-postnatal-than-prenatal maternal depressive symptoms were associated with lower right frontal FC in 18 month olds (Table 2, Fig 2e). The above findings remained unchanged when additionally controlling for the severity of maternal depressive symptoms averaged across pregnancy and the early postnatal period, monthly household income, and sleep condition during the EEG recording.

#### Externalizing and Internalizing Behaviors

There were no associations between the EPDS fluctuation and CBCL externalizing and internalizing behaviors of infants at 24 months of age (Table 2). These findings remained unchanged even after additionally controlling for the severity of maternal depressive symptoms averaged across pregnancy and early postnatal period, monthly household income, and sleep condition during the EEG recording.

### Gender Differences

The significant findings shown in the full sample (see Table 2) were also observed in the female sample but only partially observed in the male sample. Briefly, in the female sample, greater-postnatal-than-prenatal maternal depressive symptoms were significantly associated with greater right frontal activity (β **=** -0.262, p = 0.020, df = 72) and greater relative right frontal asymmetry in infants at 6 months of age (β **=** -0.426, p<0.001, df = 72) after adjusting for post-conceptual age on the EEG visit day. Moreover, greater-postnatal-than-prenatal maternal depressive symptoms were associated with lower right frontal FC in 18 month olds (β = -0.382, p = 0.002, df = 58), after adjusting for post-conceptual age on the EEG visit day. Finally, greater severity of maternal depressive symptoms predicted higher externalizing (β = 0.435, p = 0.002, df = 51) and internalizing scores (β = 0.502, p<0.001, df = 51) at 24 months of age after adjusting for maternal ethnicity and prenatal smoke exposure.

Nevertheless, in the male sample, we only observed greater severity of maternal depressive symptoms predicted higher internalizing scores (β = 0.317, p = 0.047, df = 43) at 24 months of age, after adjusting for maternal ethnicity and prenatal smoke exposure.

#### Prediction of Externalizing and Internalizing Behaviors Using Infants’ Frontal Function

**Fig 3:**
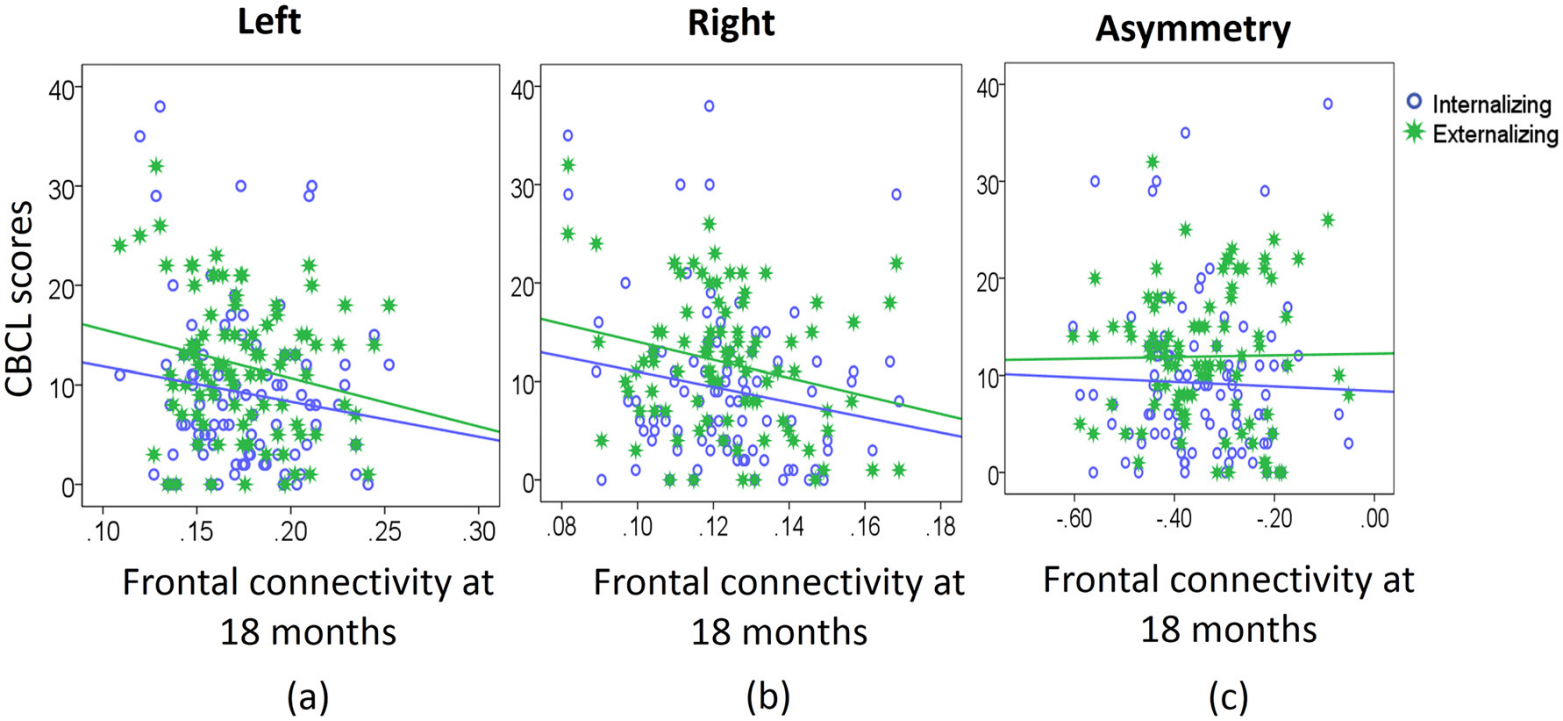
Scatter plots of child behaviors and functional connectivity. Panels show scatter plots of the externalizing and internalizing behaviors at 24 months of age against frontal connectivity at 18 months of age.

After adjusting for post-conceptual age on the EEG visit day and birth weight, neither the 6-month frontal activity nor frontal FC predicted CBCL externalizing and internalizing scores of infants at 24 months of age (Table 3). Likewise, the 18-month frontal activity did not predict CBCL externalizing and internalizing scores of infants at 24 months of age (Table 3). Interestingly, lower bilateral frontal FC at 18 months predicted a greater CBCL externalizing score at 24 months of age (Table 3; Fig 3). Lower 18-month right frontal FC was marginally associated with a greater CBCL internalizing score (β = -0.200, p = 0.066) (Table 3; Fig 3). Frontal FC asymmetry at both 6 and 18 months did not predict the CBCL externalizing and internalizing scores at 24 months of age (Table 3).

**Table 3:** The prediction of 6 and 18 months frontal EEG neural activity and functional connectivity to the externalizing and internalizing behaviors at 24 months of age.

	Power			Functional Connectivity		
	FL	FR	FA	FL	FR	FA
**6 months of age^a^**						
** Externalizing**	-0.114	-0.158	-0.097	-0.092	-0.121	-0.085
** Internalizing**	-0.005	0.000	0.021	-0.110	-0.110	-0.037
**18 months of age^b^**						
** Externalizing**	0.010	0.016	-0.001	-0.215*	-0.257*	0.002
** Internalizing**	0.062	0.016	-0.080	-0.142	-0.200	-0.048

Standardized beta values are listed in the table.

*p <0.05.

^a^Model adjusted for post-conceptual age at the 6-month EEG visit and birth weight;

^b^Model adjusted for post-conceptual age at the 18-month EEG visit and birth weight.

Abbreviations: FL–frontal left; FR–frontal right; FA–frontal asymmetry.

## Discussion

This study employed a large longitudinal normative Asian sample and investigated how pre- and early post-natal maternal depression as well as the change between them impacted infant frontal EEG function at 6 and 18 months of age and behaviors at 24 months of age. Our study did not reveal any relationships between prenatal (or postnatal) maternal depression with frontal EEG activity nor frontal connectivity of infants at 6 months and 18 months of age. Interestingly, however, an increase in depressive symptoms from the prenatal to postnatal time period predicted greater right frontal activity and relative right frontal asymmetry at 6 months of age but not at 18 months of age. An increase in depressive symptoms from the prenatal to postnatal time period also predicted lower right frontal connectivity at 18 months of age but not at 6 months of age. Furthermore, both prenatal and postnatal maternal depressive symptoms independently predicted children’s externalizing and internalizing behaviors at 24 months of age. These findings were dominantly seen in girls but not in boys.

Although relative right frontal EEG asymmetry was widely reported to reflect a vulnerability to maternal depression [3], our study found that neither pre- nor early post-natal maternal depressive symptoms independently contributed to the prediction of the right frontal activity, FC, nor their asymmetry in 6- and 18-month infants. Previous studies commonly focused on mothers with clinical levels of depression [8]. These differences likely explain the absence of an effect of maternal depression in the full sample. In our study, 216 out of 249 women (prenatally) and 184 out of 206 (postnatally) had EPDS scores less than 13, the cutoff for depression [39], and the birth weight of all infants was in the normal range. Nevertheless, consistent with past research, our study revealed independent contributions of prenatal and early postnatal maternal depressive symptoms to internalizing and externalizing behaviors at 2-years [24, 40, 41]. Another possible explanation might be because of the wide distribution of household income in our study. When including prenatally and postnatally depressed mothers with middle and upper SES, Lusby et al. (2014) found no association between postnatal depressive symptoms and the frontal EEG asymmetry at 3- and 6-month infants after adjusting for prenatal maternal depression [12].

Although pre and post-natal maternal depressive symptoms did not independently predict infant frontal function, our study demonstrated a prominent association of the change of maternal depressive symptoms from the prenatal to postnatal period with right frontal EEG activity and relative right frontal EEG asymmetry in 6-month infants, that is, increasing maternal depressive symptoms from the prenatal to postnatal period predicted greater right frontal activity and relative right frontal asymmetry amongst 6-month infants. This showed a similar trend to the previous finding derived from the GUSTO sample using structural magnetic resonance imaging (MRI) data [42]. We previously found that increasing maternal mood symptoms from the prenatal to postnatal period predicted faster growth of the right hippocampus of infants over the first 6 months of life. Together, these findings suggest that the anatomical and functional development of individual brain structures in the fronto-limbic circuit is likely to reflect the influence of both pre- and early post-natal maternal mood, especially the fluctuation in level of maternal mood from pre- to early post-natal period. Such findings are important, since the frontal region has been considered as a key neural locus of psychopathology [43], including depression in children [44], adolescents [45], and adults [46].

Our study provided novel evidence of the association between the fluctuation of maternal depressive symptoms from the prenatal to postnatal period and frontal FC of 18-month infants. Alterations in functional connectivity have been increasingly recognized as a marker for various psychiatric disorders, including major depressive disorder [27–29]. We did not find significant effects of the fluctuation of maternal depressive symptoms on FC at 6 months, however, we did observe a negative relationship between the fluctuation of maternal depressive symptoms and the right frontal FC at 18 month. This may suggest a pathway through which maternal depressive symptoms, over time, first influence neural activity in terms of the EEG activity in earlier age (6-month olds), and ultimately disrupt the functional integration of infant brain in later age (18-month olds). Such a progressive influence of maternal mental health on infants’ frontal function and functional connectivity is in synchrony with the development of the frontal region. A postmortem brain study showed that the emergence of structural connectivity moved generally from posterodorsal to anteroventral regions in infants aged between 17 to 40 weeks post-conception [47]. Diffusion tensor imaging (DTI) studies have also showed greater development of axonal connections in the frontal lobe from the 10 months to 24 months post-conceptual period than at earlier time points [48]. Taken together, the influence of maternal mental health on the infant frontal function and connectivity is progressive and synchronized with the development of brain anatomy and function and their organization.

Furthermore, it has been suggested that both pre- and post-natal maternal stress can alter neurodevelopment of the offspring [9, 24, 49]. Prenatal maternal depression may alter offspring neurodevelopment by increasing fetal exposure to maternal cortisol [9]. That is, the placental enzyme 11β-hydroxysteroid dehydrogenase type 2 (11β-HSD2), which serves as a partial barrier, may be downregulated by an increase in maternal cortisol such that a proportion of maternal cortisol passes through the placenta [50], resulting in alterations in receptor expression in areas accompanying changes in neural development [51]. After birth, maternal depression may affect parenting practices and parent-child relationships, which lead to an increase in children’s perceptions of threat and the production of endogenous stress hormones [13, 15]. Together with our findings, these findings highlight that stress fluctuation before and after delivery may be important for the neurodevelopment in early life. Infant mental health studies have shown that infants’ brains develop at an astonishing rate in the first two years of life, with dendritic and synaptic proliferation in response to environmental stimulation. Pathways that are activated repeatedly are enhanced, whilst those not required are pruned [52]. In infants exposed to psychosocial stress and deprivation, the primitive limbic pathways, remain over-activated as the infant has to respond with over-drive of the stress response [53]. This depletes resources and attention from higher functioning cortical development [54], evidence similarly supported in our sample of lower frontal functional connectivity. The resultant developmental trajectory is thus towards over-driven behavioral responses required for survival in the young child, such as aggression and withdrawal[55].

Supporting this theory of stress-driven neurodevelopment, our study observed the relationships between the frontal FC and externalizing and internalizing behaviors in early life. In particular, our study showed that lower bilateral frontal FC at 18 months of age predicted higher externalizing behavioral scores, while lower right frontal FC predicted higher internalizing behavioral scores in children at 24 months of age. Hence, our results highlighted the importance of frontal FC in the prediction of internalizing and externalizing problems. However, our study did not reveal any significant associations of the frontal EEG activity with internalizing and externalizing behaviors. In addition, 10-year-old children with greater relative right frontal EEG neural activity (lower relative right frontal power) exhibited higher externalizing behaviors [56]. Nevertheless, a meta-analysis study also suggested that the frontal EEG asymmetry showed considerably weak or non-significant relation to internalizing and externalizing symptoms [3]. Compared to the frontal FC measure, the frontal EEG activity measure had less statistical power in predicting children’s behaviors at 24 months of age in our study. Therefore, over the time of the brain development, our observations supported growing evidence that the organization of the brain’s functional network might be parallel to the behavioral development, even in early postnatal life.

Not surprisingly, our findings were predominantly observed in girls but not in boys. This is largely consistent with literature. Increasing evidence suggests that girls are more susceptible to the influences of maternal depression than boys are [57]. Girls may be sensitive to elevated levels of stress and maternal depression is a nonspecific stressor that may increase the risk already posed to girls [58, 59]. The heritability for depression in girls was higher than that in boys [60].

Our study has several strengths. It was conducted with, a large normative Asian sample in a longitudinal manner, and included EEG activity and functional connectivity in relation to pre- and post-natal maternal depressive symptoms. However, there were several limitations in our study. Our assessment of maternal depressive symptoms was based on a common screening tool designed to elicit a subjective report of emotional well-being, but not a clinical diagnosis. In addition, although we assessed both pre- and post-natal depression, due to considerations of subject burden, we were limited to assessing depression at only 26 weeks gestation and 3 months postpartum. Nevertheless, it is important to note that the second and third trimesters of pregnancy are critical periods when neural migration and synaptogenesis of the fetal brain occurs. In addition, we did not incorporate the scale of later postnatal maternal depressive symptoms into the analysis as the scales of the 3-month (mean±SD: 6.8 ± 4.8) and 24-month EPDS (mean±SD: 6.5 ± 5.1) were not statistically different (Paired Sample t = 0.739, p = 0.461). Last but not least, our study did not incorporate potential influences of early maternal behavior and genetic variations on the brain development, which requires further investigation.

In conclusion, our study utilized a large longitudinal Asian sample and provided the first evidence on progressive influences of the fluctuation of maternal depressive symptoms from pregnancy to early postnatal period, first on the EEG activity of the frontal region and then on its functional integration across the brain in the later stage. Such a progressive influence may suggest that the fluctuation of maternal depression has a long-term impact on the development of the brain functional organization in later life. Furthermore, altered frontal FC in infants born to mothers with higher fluctuation of a maternal depression score from pre- to post-natal period may reflect a neural basis for the familial transmission of phenotypes associated with psychopathology. In addition, these findings suggest that studying frontal functional integration assessed using functional connectivity may enhance our understanding of the role of the frontal region in the development of mental illness in early life. Last but not least, our study supports the dimensional view of maternal depressive symptoms, that is, symptoms of maternal depression lie on a continuum. Hence, even though maternal depressive symptoms are in a normative range, they could still influence the brain development of the offspring.
